# Ion Channels and Zinc: Mechanisms of Neurotoxicity and Neurodegeneration

**DOI:** 10.1155/2012/785647

**Published:** 2012-05-07

**Authors:** Deborah R. Morris, Cathy W. Levenson

**Affiliations:** ^1^Department of Biomedical Sciences, Florida State University College of Medicine, Tallahassee, FL 32306 4300, USA; ^2^Program in Neuroscience, Florida State University College of Medicine, Tallahassee, FL 32306 4300, USA

## Abstract

Ionotropic glutamate receptors, such as NMDA, AMPA and kainate receptors, are ligand-gated ion channels that mediate much of the excitatory neurotransmission in the brain. Not only do these receptors bind glutamate, but they are also regulated by and facilitate the postsynaptic uptake of the trace metal zinc. This paper discusses the role of the excitotoxic influx and accumulation of zinc, the mechanisms responsible for its cytotoxicity, and a number of disorders of the central nervous system that have been linked to these neuronal ion channels and zinc toxicity including ischemic brain injury, traumatic brain injury, and epilepsy.

## 1. Introduction

Although zinc is clearly an essential trace element that is required for the function of hundreds of enzymes and DNA-binding transcription factors, excessive zinc has long been implicated in processes leading to cellular damage. In the central nervous system large amounts of zinc can enter postsynaptic neurons through a variety of ion channels including glutamate receptors and voltage-gated calcium channels. This paper will first discuss the cellular pools of zinc, the mechanisms that appear to be responsible for the accumulation of neuronal zinc, and then outline the current hypotheses about how excess zinc exploits ion channels and other mechanisms to produce neurotoxicity in the hippocampus, amygdala, and cortex under pathological conditions. With these mechanisms in mind we will then discuss relevant clinical situations such as traumatic brain injury, ischemic injury (stroke), and epilepsy, where excess zinc accumulation can lead to neurodegeneration.

## 2. Neurotoxic Zinc: Cellular Sources and Routes of Entry

### 2.1. Sources of Neurotoxic Zinc

 While a majority of zinc in the central nervous system (CNS) is tightly bound to zinc-dependent enzymes and other proteins, approximately 10% is “free” or “chelatable” zinc which is not associated with proteins or aminoacid ligands. Under pathological conditions, free zinc appears to participate in the neurotoxic accumulation of zinc in neurons. In normal neurons, free zinc is predominately localized to the presynaptic vesicles of glutamatergic neurons [1, 2]. Free zinc has also been colocalized to GABA and glycine containing murine neurons [3]. Regions rich in vesicular free zinc include the mossy fibers of the hippocampus, the amygdala, and the olfactory bulb. “Zincergic” neurons are also abundant in the cortex [4].

 In addition to the large pool of vesicular zinc, there is clear evidence for additional intracellular pools of zinc that can be liberated to form free zinc. The strongest case for the presence of nonvesicular pools of free zinc comes from a report showing that free zinc accumulates after seizure activity in animals that lack vesicular zinc [5]. This work measured free zinc accumulation in the hippocampal neurons of ZnT3-null mice that lack the ability to pump zinc into synaptic vesicles. While this work did not definitively determine the source of the non-vesicular free zinc, the surprising finding that these animals exhibited accumulation of free zinc in damaged neurons after kainate-induced seizures began the hunt for alternative pools of free zinc that may participate in neurotoxicity. Subsequently, others have identified a mitochondrial pool of zinc that can be both influenced by the amount of intracellular zinc as well as contribute to it [6]. There is also now evidence that protein bound zinc can be mobilized to form a free zinc pool under oxidative conditions [7].

### 2.2. Routes of Neurotoxic Zinc Entry

Upon neuronal excitation, vesicular zinc is released into the synaptic cleft. Under normal conditions, the primary function of the zinc from synaptic vesicles appears to be the modulation of both ionotropic and metabotropic post-synaptic receptors through zinc-specific allosteric binding sites. For example, zinc inhibits GABA_A_ receptors, reducing their inhibitory action [8, 9]. The effect of zinc on excitatory glutamate receptors is complex. Not only can zinc act as an inhibitory neuromodulator of glutamate release [10], but it was initially thought to inhibit activity of NMDA glutamate receptors [9, 11]. However, there are reports of biphasic and cell type-specific zinc regulation of both NMDA and AMPA/kainate glutamate receptors [12–15]. Additionally, zinc can potentiate glycine-mediated currents [16] and regulate voltage-gated calcium channels [17] as well as potassium, sodium, and chloride channels [18].

However, under pathological conditions, excess free zinc is released from synaptic and other free zinc pools. As excess zinc floods the synaptic cleft, it exploits a variety of receptors and channels to gain entry into post-synaptic neurons. There appear to be at least four different routes of entry. First, AMPA/kainate glutamate receptors have been identified as the primary route of entry for zinc into post-synaptic neurons [19]. Zinc also exploits NMDA glutamate receptors to gain entry into neurons. Both of these glutamate binding receptors transport calcium as well as zinc into post-synaptic neurons. The third route of entry for toxic levels of zinc is voltage-gated calcium channels [19–21]. Finally, excess zinc appears to enter neurons via a transporter-mediated exchange with intracellular sodium. While the presence of a putative Na^+^/Zn^+2^ exchanger has been hypothesized, it is also probable that when excess zinc is released from presynaptic neurons, it can replace calcium and gain entry into neurons via Na^+^/Ca^+2^ exchange proteins [20].

## 3. Mechanisms of Zinc Neurotoxicity

 Damage to the CNS induced by seizure, trauma, or ischemia can all result in the accumulation of zinc from vesicular and non-vesicular zinc pools. While it is clear that the excess zinc that is detected in these and other pathological conditions is neurotoxic, there is considerable debate about the cause of the toxicity. There are three main hypotheses that are currently being explored, namely, that excess zinc: causes excitotoxicity, induces oxidative stress, and impairs the generation of cellular energy. In fact, as the next several sections will show, there is evidence for each of these mechanisms, suggesting that they are not mutually exclusive, and that all three actions of zinc may be acting synergistically to cause neuronal damage and death.

### 3.1. Excitotoxicity

The influx of excess zinc into neurons has been shown to result in excitotoxic damage to post-synaptic neurons. This is in part mediated by the action of zinc on glutamate receptors and other ion channels. For example, when large amounts of glutamate and zinc are released together from pre-synaptic neurons, there is an acute block of NMDA glutamate receptors as zinc binds to allosteric sites on these ion channels. However, this acute downregulation is followed by a Src family kinase-mediated upregulation of NMDA receptor activity that appears to participate in neuronal damage and cytotoxicity [12].

### 3.2. Oxidative Stress

Excess zinc has been implicated in the generation of oxidative stress by free radicals and reactive oxygen species (ROS) leading to neuronal damage and death. In fact, it was recently hypothesized that the combination of age-related oxidation and resulting zinc accumulation could act synergistically to promote aging-related neuronal damage and neurodegeneration [22]. In support of this hypothesis, treatment of mouse cortical neuron cultures with 30–35 *μ*M zinc for 24 hours triggered processes eventually leading to neuronal, but not glial, death [23] that was accompanied by membrane lipid peroxidation. Interestingly, while the neurotrophins NT-3, NT-4/5 and brain-derived neurotrophic factor (BDNF) were unable to prevent the zinc-induced neurotoxicity, the water soluble form of vitamin E, trolox, almost completely prevented the neuronal death associated with excess zinc in these cultures [23, 24]. Zinc toxicity, both as a result of zinc applied directly to neuronal cultures and after kainate application, was also prevented by the addition of glutathione [25]. Together, these data suggest that much of the neuronal damage was the result of free radical generation.

It should be noted that the neuronal death was accompanied by classical features of apoptosis including internucleosomal DNA fragmentation, as well as other characteristics commonly associated with necrosis such as swelling of intracellular organelles [23, 24]. While the authors of this early report suggest that zinc may trigger necrosis, subsequent work has shown that neuronal apoptosis is complex and frequently cannot be categorized into strictly apoptosis or necrosis, but is probably best described as a continuum, with characteristics that include those classically associated with both apoptosis and necrosis [26].

Clearly the next question is *how* excess zinc leads to the production of ROS. Early evidence suggested a role for zinc in the regulation of neuronal protein kinase C (PKC) [27]. While this initial report linked the zinc-regulation of PKC to oxidative damage, it was subsequent work that led to the elucidation of the mechanism responsible for the PKC-dependent generation of ROS in zinc-loaded neurons. Namely, it appears that zinc-stimulated PKC enhances the expression of the enzyme NADPH oxidase. Zinc also facilitates the translocation of the NADPH oxidase subunits p47^PHOX^ and p67^PHOX^ to the neuronal membrane, where they participate in the generation of ROS [28]. Zinc-stimulated NADPH oxidase may also stimulate the activation of poly (SDP-ribose) polymerase (PARP), which can trigger apoptotic processes [29]. While it is clear that these may not be the only mechanisms responsible for the generation of ROS in zinc-loaded cells, the work does shed light on the role of oxidation in zinc toxicity.

### 3.3. Impaired Energy Production

While early work showed that nanomolar concentrations of zinc were capable of significantly inhibiting the glycolytic enzyme glyceraldehyde-3-phosphate dehydrogenase (GAPDH), our current understanding of the role of zinc in neuronal glycolysis is that excess zinc rapidly reduces cellular levels of nicotinamideadenine dinucleotide (NAD^+^) [30–32]. This reduction in NAD^+^ is not only responsible for the reduced activity of GAPDH and the subsequent accumulation of dihydroxyacetone phosphate (DHAP) and fructose 1,6 bisphosphate, but also leads to a reduction in ATP levels and subsequent neuronal death [30]. Reductions in NAD^+^ would be expected to reduce cellular ATP production not only because it inhibits the activity of glycolytic enzymes, but also because of the resulting inhibition of mitochondrial respiratory enzymes. In fact, several studies have reported reductions in the NAD^+^ dependent enzymes alpha-ketoglutarate dehydrogenase and isocitrate dehydrogenase as well as other mitochondrial enzymes including succinate dehydrogenase and aconitase [33, 34].

Other mechanisms leading to zinc-induced mitochondrial dysfunction including the RAS/ERK/MAPK signaling pathway [35], where ERK 1/2 is likely activated via zinc induction of the immediate early gene *erg-1* [36], and MAPK signaling leads to mitochondrial hyperpolarization [35]. Furthermore, mitochondrial trafficking, which has been hypothesized to play a role in neurodegeneration, is impaired by excess zinc, potentially by a phosphatidylinositol 3-kinase-mediated mechanism [37]. Not only can zinc act as an independent modulator of mitochondrial function, it also appears to act synergistically with other agents such as kainate to induce mitochondrial dysfunction and neuronal death [38, 39]. This appears to be facilitated by the mitochondrial uptake of zinc, followed by the release of proapoptotic mitochondrial proteins such as cytochrome c and the apoptosis induction factor, AIF [40].

## 4. Zinc Toxicity in Neurological Disease and Disorders

The proposed mechanisms associated with zinc neurotoxicity have been implicated in a large number of neurodegenerative disorders including amyolateral sclerosis, Alzheimer's disease, and Parkinson's disease. The strongest links, however, between the excess zinc release and uptake via ion channels have been observed in models of ischemic brain injury, traumatic brain injury, and seizure disorders.

### 4.1. Zinc and Ischemic Brain Injury

Although its role is under active investigation, zinc dysregulation has been implicated in mechanisms leading to neuronal damage and death after ischemic brain injury. Tønder et al. first suggested a role of zinc toxicity in the neuronal death associated with global ischemia. In this early report, when adult rats were subjected to global brain ischemia there was a decrease in free zinc associated with mossy fiber layers of the hippocampus and an increase in the dentate hilar region of degenerating neurons [41]. In another model of stroke, transient forebrain ischemia, zinc left pre-synaptic terminals and accumulated in post-synaptic neurons. This translocation was associated with degeneration of neurons in the hippocampus, cerebral cortex, thalamus, striatum, and amygdala. An intracerebral injection with the membrane impermeant zinc chelator Ca-EDTA prior to ischemia was neuroprotective and significantly decreased neuronal death [42]. Administration of Ca-EDTA 30 min prior to mild focal ischemia reduced infarct size 3 days after injury. However, the infarct reduction was lost when rats were euthanized 14 days later, even when Ca-EDTA was continuously perfused [43].

Aspirin is an anti-inflammatory drug commonly used for secondary prevention after stroke and is protective against NMDA-induced and zinc-induced neurotoxicity. The application of 3–10 mM aspirin to cortical neurons exposed to 300 *μ*M zinc for 30 min prevented neuronal degeneration but not oxidative stress or apoptosis. It was shown that aspirin prevented zinc entry into neurons by inhibiting voltage-gated calcium channels, which is a main route for zinc entry into neurons [44].

### 4.2. Zinc and Traumatic Brain Injury

There is also a relationship between accumulation of free zinc and neuronal degeneration following traumatic brain injury. In one study, adult rats and neonatal pups were given a unilateral cortical stab wound. Four weeks following injury, adult rats, but not neonatal pups, showed increased TSQ staining of free zinc in and around the site of injury, suggesting that the accumulation of free zinc is responsible for neuronal death [45]. A TBI model of mechanical cortical trauma in rats resulted in loss of zinc from pre-synaptic vesicles and movement into post-synaptic neurons accompanied by neuronal death in the hilus, dentate gyrus, and CA1 regions of the hippocampus [46]. It was further demonstrated that rats given a mild or moderate TBI by a fluid percussion injury showed that injured neurons stained with Fluoro-Jade in the hippocampal regions of CA1, CA3, and dentate gyrus 4 hours and 24 hours after injury contained high levels of free zinc as detected by the zinc indicator Newport Green. This is an important finding because it suggests that an increase in free zinc after brain injury is neurotoxic and causes neuronal death in the hippocampus [47].

Additional evidence that high levels of free zinc are involved in neuronal degeneration after brain injury comes from experiments demonstrating that injections of the extracellular zinc chelator Ca-EDTA before TBI provided a neuroprotective effect by reducing cell death in the hippocampus [46]. Pretreatment with Ca-EDTA before a TBI-induced model of fluid percussion injury reduced apoptotic cell death measured by TUNEL labeling while also upregulating the expression of neuroprotective genes [48]. However, rats that were given a TBI and Ca-EDTA treatment did not have improved learning and memory performance in the Morris Water Maze test two weeks after TBI. Although zinc chelation provides short-term histological benefits, it does not appear to improve long-term functional outcomes [49].

Interestingly, recent evidence has suggested that the source of the zinc that accumulates after TBI in mice is not exclusively vesicular and has led researcher to question some of the previous assumptions about the role of zinc in brain injury. ZnT3-knockout mice that lack vesicular zinc showed an increase in injured neurons and apoptotic cells compared to wild-type (WT) control mice 24 hours after injury. After chemical blocking of vesicular zinc in post-TBI ZnT3 KO and WT mice, damage was unchanged in ZnT3 KO mice, while the numbers of apoptotic cells increased in WT mice with levels comparable to ZnT3 KO mice. This new evidence suggests that free vesicular zinc may actually play a neuroprotective role after TBI [50]. Clearly more work will be needed to understand the role of zinc, glutamate receptors, calcium channels, and other mechanisms after traumatic brain injury. In light of these new data it is interesting to note that administration of zinc appears to have a positive effect on outcomes after TBI. In one clinical trial, zinc supplementation improved neurological scores following TBI [51]. More recent work in a rat model of TBI showed that zinc can provide behavioral resiliency to TBI when given 4 weeks prior to injury and resulting in improved cognition and reduced depression- and anxiety-like behaviors [52]. The mechanisms responsible for the robust effect of zinc in this model are currently under investigation ZnT3.

### 4.3. Zinc and Epilepsy

Zinc dysregulation and homeostasis has been suggested to play a role in the development of seizures. In an epilepsy mouse model, zinc levels were significantly lower in the hippocampus when compared to control mouse strains [53]. There is also evidence that zinc translocates from pre-synaptic boutons into post-synaptic neurons, possibly causing neuronal degeneration after seizure [54].

As discussed previously, synaptic zinc acts as a neuromodulator and regulates the activity of a variety of post-synaptic receptors including NMDA, GABA_A_, and AMPA receptors, which then determine neuronal excitability [55]. As a result of its ability to act as a neuromodulator, zinc has been implicated as a proconvulsant [56] and anticonvulsant [57]. Studies that support the proconvulsant theory showed that seizures were induced by an injection of zinc sulfate in rabbits [56]. It has also been shown that zinc chloride enhances kainate neurotoxicity in the hippocampus. Interestingly, CNQX, an NMDA antagonist, helped to prevent neuronal damage [58]. During kindling-induced seizures in which a stimulus was applied for ten times a day for two days, and the zinc chelator diethildythiocarbmate (DEDTC) was injected before each stimulus, duration of behavioral seizures and electrical discharges were decreased [59].

Despite this work, most data support the idea of zinc as an anticonvulsant. Increased susceptibility to kainate-induced seizures was observed in both ZnT3 KO mice lacking vesicular zinc [55] and in mice fed a zinc deficient diet for 4 weeks [60]. After kainate treatment, hippocampal extracellular fluid in zinc deficient mice had increased levels of glutamate and decreased levels of GABA compared to control animals [60]. Furthermore, in a mouse model of epilepsy, seizure susceptibility was increased by zinc deficiency and reduced by zinc supplementation [61]. Chelation studies further support the anticonvulsant theory in that membrane permeable and membrane impermeable zinc chelators have little effect on seizure activity in the CA3 region of the hippocampus [62].

Since most studies suggest that zinc plays an anticonvulsant role in seizures, it is important to look at the possible role of zinc supplementation as a therapeutic agent in the treatment of seizure. The administration of zinc directly into the dentate gyrus of the hippocampus delayed behavioral seizures during electrical stimulation in adult Wistar rats [63]. Intraperitoneal injections with a medium dose of zinc (3 mg/kg) and with the antiepileptic drug valproic acid either alone or in combination reduced the severity of pilocarpine-induced seizures. In contrast, high doses (60 mg/kg) of zinc exacerbated the severity of seizures highlighting the dose-dependent effect of zinc [64].

## 5. Conclusion

 Neuronal ion channels and receptors and zinc toxicity appear to play a role in a number of neurological disorders and diseases. Gaining access to neurons through these receptors and channels, excess zinc leads to excitoxicity, oxidative stress, and impairment of neuronal energy production, all of which not only damage neurons but also lead to neuronal death. Future work will be needed to develop strategies to block zinc-mediated damage and prevent poor outcomes associated with stroke, traumatic brain injury, and epilepsy as well as other neurodegenerative disorders that may respond to therapies designed to modulate ion channels and zinc.
